# Exploring the use of health technology in community-based midwifery care – an interview study

**DOI:** 10.1186/s12884-025-07406-5

**Published:** 2025-03-14

**Authors:** Holly Edmundson, Margaret Glogowska, Gail Hayward, Jude Mossop

**Affiliations:** 1 Nuffield Department of Primary Care Health Sciences, University of Oxford, Radcliffe Observatory Quarter, Woodstock Road, Oxford, OX2 6GG UK; 2 Nuffield Department of Women’s & Reproductive Health, University of Oxford, Level 4, Women’s Centre, John Radcliffe Hospital, Oxford, OX3 9DU UK

**Keywords:** Community midwifery, Health technology, Point-of-care testing (POCT), Maternity care, Midwifery equipment, Digital health, Diagnostic technology, Community healthcare, Clinical decision-making, Workforce challenges

## Abstract

**Background:**

New portable health technologies may offer solutions to challenges in current maternity care, but little is known about their current usage, existing problems, or areas of unmet needs.

**Purpose:**

To better understand the use of health technology in community midwifery care in the UK.

**Methods:**

Midwives with current or recent experience working in community settings were recruited using social media. Semi-structured interviews were undertaken. These were transcribed and thematically analyzed.

**Results:**

Thirteen midwives were interviewed between October 2021 and March 2022. The main themes and subthemes were: (1) Problems with current equipment: (a) Issues in the context of remote working, b) Concerns regarding accuracy, and c) Midwives’ perceptions of service user experiences. (2) Equipment challenges working within the UK National Health Service: (a) Lack of availability of appropriate equipment, and (b) Lack of autonomy in how to utilize equipment. 3) Areas of unmet needs.

**Conclusion:**

This study has shown that there are several areas of unmet needs for community midwives that should be investigated. However, and arguably more pressing, is improving the availability and quality of health technologies and other equipment that is already widely used. Midwives were interested in technologies that support decision making, reduce the number of hospital visits for their patients, improve their workload, and reduce medicalisation. At the same time, there is fear that technology may displace midwives’ wisdom. Where new technologies are introduced, support and training should be provided to address potential resistance.

## Introduction

Midwifery training in the UK was formalized with the introduction of the Midwives Act in 1902, which included guidance on the equipment that midwives should carry [1]. Much of the equipment used today, including handheld fetal dopplers [2] and urinalysis sticks [3] was first introduced in the mid-20th century. Updating midwives’ equipment has been slow, despite significant developments in portable health technology, sometimes referred to as point-of-care tests (POCTS), that enable hospital-level diagnostics in the home and community [4].

The National Health Service (NHS) Maternity services in the UK are under enormous pressure. Recent independent enquires and surveillance programs have highlighted failings in maternity services that must be addressed [5]. In the context of significant staff shortages and increasingly complex caseloads this is not a simple task. Introduction of novel health technologies in community midwifery settings has the potential to enhance improvement strategies [4]. For example, such efforts may relieve pressure in obstetric units and make needed healthcare more accessible for women. In order to stimulate the use and development of appropriate medical technologies for community maternity services, it is important to review the current experience of midwives. This study aims to understand their knowledge, experiences, and perceptions of health technologies, including their views on the areas of unmet needs.

## Methods

This study uses a qualitative approach to understand the lived experiences of community midwives in the UK regarding health technologies. By emphasizing individual experience, this orientation aims to provide authentic insight into the equipment challenges midwives face [6]. Qualitative research captures and explores people’s experiences and perceptions [6]. Therefore, qualitative semi-structured interviews were used to explore midwives’ knowledge, experiences, and perceptions of health technologies used in community maternity care. This method allows researchers to cover issues of interest to them but also allows the participants to voice issues of personal and professional significance and importance [7].

### Sampling and recruitment

Midwives with current or previous experience working in community-based settings within the UK were invited to participate in this study. The study was promoted on social media (on Facebook groups such as ‘Maternity and Midwifery Forum’ and ‘Continuity Matters’ and ‘Midwifery Study Days & Conferences’ and on Twitter) with posts linking to the study webpage. This webpage included the Patient Information Leaflet (PIL) and contact details for the study team for those interested in taking part. All those who responded and met the inclusion criteria were invited to participate in the interview. The decision to stop interviewing was discussed and agreed upon among the members of the research team when sufficient information had emerged, and there was a satisfactory explanation of the emerging themes.

### Interviews

Qualitative semi-structured individual interviews lasting approximately 30 min were conducted by telephone. The interviews were all performed by the primary researcher, HE, apart from one, done by JM. Informed verbal consent was obtained prior to the interview. A topic guide was used. The topics were based on the available literature and drew on issues from topic guides for other studies that have investigated clinicians’ views of POCTs [8, 9]. When developing the topic guide for a midwifery audience, informal feedback was obtained from community midwives. The guide was amended and added to as the interviews progressed.

The interviews were recorded using a digital audio recorder and transcribed verbatim by a professional transcriber. The primary researcher made field notes during and after the interviews.

### Analysis

The transcripts were checked for accuracy and anonymised before being uploaded to a specialist software programme to assist in the organization and analysis of data (NVivo V.1). Thematic analysis of all the interviews was performed using a grounded-theory approach [10]. Grounded Theory is a qualitative methodology which guides systematic data collection and data analysis. This approach enables iterative generation of information which is grounded in the data through constant comparison. It is a particularly useful approach when exploring new or under-researched topics. Codes were used to group the data. Categories were created by grouping and comparing the codes. Single-sheet brainstorming supported the development of broad themes [11]. Transparency and dependability were ensured by creating an audit trail from the raw data to the themes and seeking the agreement of the analysis from the research team [12].

### Researcher characteristics and reflexivity

The primary researcher was HE (MSc, female), a practicing community midwife and early career researcher. JM (BA (Hons), female) is a Senior Research Midwife with experience in qualitative research. MG (PhD, female) is a senior qualitative researcher. MG supported HE throughout the data collection and analysis process, including providing interview training. The midwives interviewed were given no information about HE or JM, apart from their current job titles. One of the midwives interviewed was known to HE, as they worked at the same NHS Trust but on different teams. Prior to the interviews, the participants were in contact with HE minimally, only to schedule their interviews. Having midwife interviewers allowed for flowing conversation. At times, interviewees asked HE about her own experiences. When this happened, HE explained that these could be shared and discussed once the interview was completed.

### Findings

Thirteen interviews were conducted between October 2021 and March 2022. A further two midwives contacted the research team regarding participation. One was unable to be interviewed due to unavailability and the other did not meet the eligibility criteria.

### Participant characteristics

All of the participants were midwives who were working in community settings within the National Health Service (NHS) or who had done so in the 12 months preceding the interview (Table 1).

**Table 1:** Participant characteristics

	Work setting	Years qualified
Midwife A	Suburban	5–10
Midwife B	Urban and Suburban	10–20
Midwife C	Rural	10–20
Midwife D	Rural	10–20
Midwife E	Rural	< 5
Midwife F	Rural	10–20
Midwife G	Rural	10–20
Midwife H	Suburban	> 20
Midwife I	Urban	< 5
Midwife J	Suburban and rural	< 5
Midwife K	Urban and Suburban	5–10
Midwife L	Urban	5–10
Midwife M	Urban	10–20

### Health technology used by the midwives interviewed

The midwives described using health technology for a range of tasks, including assisting in clinical decision-making, supporting public health initiatives, and providing reassurance. The health technologies used in a community setting by at least one of the midwives interviewed in this study are shown in Table 2. There are currently very few in vitro Diagnostic (IVD) POCTs (equipment that quickly analyses samples taken from the human body - immediately, on-site) used in community midwifery practice. The midwives interviewed did collect samples but would then send them to a hospital laboratory for analysis. Similarly, ultrasound scanning in the midwives’ NHS Trusts was performed exclusively within a hospital, and not by community midwives.

**Table 2:** Health technologies currently used by study participants

Name	Assessment	Other information
Sphygmomanometer (‘sphyg’)	Maternal blood pressure	Midwives described using manual and digital versions.
Urinalysis sticks	Pathological changes in urine (pre-eclampsia, gestational diabetes, urinary tract infection).	Midwives described using sticks testing Proteinuria only, or Glucosuria and Proteinuria only, or sticks with up to 10 tests including proteinuria and glycosuria.
Carbon Monoxide Monitor	Carbon Monoxide level	Used to identify women who may be smoking in pregnancy and supporting those who are quitting.
Handheld fetal doppler	Fetal heart rate	Devices with audio output only or with an audio and visual output (numeric or/and trace modes).
Thermometer	Temperature (maternal and neonatal)	The midwives discussed different types including electronic versions and single use versions (strip and dot tests).
Oxygen saturation monitor	Oxygen saturations	Separate maternal and newborn monitors, both electronic.
Scales	Weight	Separate maternal and newborn scales (sling/tray). Analog and electronic.
Cardiotocograph (CTG)	Continuous monitoring of the fetal heartbeat and uterine contractions	Used as an antenatal assessment of fetal wellbeing following maternal report of reduced movements. Not used in intrapartum care by the community midwives interviewed, except in circumstances where there were concerns and they were awaiting transfer to an obstetric unit by ambulance.
Liquor detector	Assessment of rupture of membranes	Vaginal swab or sanitary pad with an indicator.
Lateral flow test	Covid-19 infection	Used in intrapartum care, sometimes routinely and sometimes in the presence of symptoms.
Transcutaneous bilirubinometer	Bilirubin level	Assessment for the presence/severity of neonatal jaundice
Equipment for serum bilirubin testing	Bilirubin level	Assessment for the presence/severity of neonatal jaundice
Ophthalmoscope	Newborn eye assessment	Used to assess presence of a ‘red reflex’

### Themes and subthemes

The main themes and subthemes identified by the research team were as follows:

1. Problems with current equipment.
  - Issues in the context of remote working.
  - Concerns regarding accuracy.
  - Midwives’ perceptions of service user experiences.
2. Equipment challenges working within the NHS.
  - Lack of availability of appropriate equipment.
  - Lacking autonomy in how to utilize equipment.
3. Areas of unmet needs.

### Theme one: 1) problems with current equipment

The midwives interviewed were generally satisfied with the health technology and equipment available to them. However, several recurrent issues emerged. Notably, despite describing serious challenges, midwives often framed these issues as mere inconveniences—suggesting a level of normalisation and acceptance of suboptimal conditions as an inherent part of their role.

#### 1a. Issues in the context of remote working

Most of the midwives interviewed worked from a central base, such as a midwifery led unit, but would deliver much of their care in women’s homes or from GP surgeries and community spaces. They transported necessary equipment in their cars, and so wanted items to be portable and sturdy. Many of the midwives highlighted handheld fetal doppler devices as being too fragile. Several described doing ‘DIY repairs’ while waiting for a new device.

*We also have a doppler*,* which is just about giving up the ghost because they’re not the most robust things in the world. It’s being held together with tape until I can get a new one.*

Midwife B.

Almost all the midwives interviewed reported issues with thermometers, including axillary, tympanic, and single-use models. The accuracy was felt to be unreliable, and there were concerns that this was exacerbated when they had to store their equipment in imperfect conditions, for example, in their cars.

*We’re not supplied with reliable equipment in the community… I think many of us have bought our own thermometers because the ones that we’ve been issued with are rubbish… We were issued some time ago with thermometers that you put under the arm and they take about 3 min to read… you can get them in the pound shop and to be honest they’re just as good. We sometimes have stocks of the tempedots [single use thermometers] and they’re okay until you’ve had them in the car in warm weather.*

Midwife M.

Many midwives also felt that the equipment provided to them was heavy or bulky, which they found impractical or potentially dangerous. Baby weighing scales, with a tray for the baby, were highlighted as a particular example. Some midwives interviewed used hanging scales, which they preferred, but most of those interviewed were not given this option.

*If there’s a way of making stuff*,* I don’t know*,* smaller or lighter or more compact or easier to transport? So it’s easier on midwives’ backs*,* because our backs go.*

Midwife A.

#### 1b. Concerns regarding accuracy

Several midwives had experiences with packs of urinalysis sticks that they thought were faulty or had degraded (for example, if the lid was left off). One midwife felt that the test took too long and suggested that this probably resulted in some underdiagnosis.

*You know on the odd occasion you’ll get a dud set [of urinalysis sticks] where literally everyone has protein*,* and then you realize that you’ve got a dud pack and you just change the pack…. Or if someone tells me they’ve got good signs of a UTI [urinary tract infection] and it’s completely clear*,* I’ll just try my other packet just in case.*

Midwife I.

Many of the midwives discussed POCTs for diagnosing pre-labor rupture of membranes. The tests, which come in various forms, including swab tests and sanitary pads, detect amniotic fluid. These were not used by all the midwives and several reported that these were introduced and then withdrawn due to concerns regarding accuracy (and in one case, cost). The midwives generally felt that these would be useful, so long as the result could be trusted.

*We did occasionally use amniopads… They’re sanitary towels that change colour in the presence of amniotic fluid. But*,* they don’t seem to be very reliable. I know of some women who had definitely SROM’d [spontaneous rupture of membranes]*,* but the pad didn’t change colour. We didn’t get them back again and I think that was why*,* because they weren’t very reliable.*

Midwife C.

#### 1c. Midwives’ perceptions of service user experiences

The midwives were sensitive to the experiences of women in their care, particularly because pregnancy and birth are times of increased physical demand and psychological vulnerability. They felt that some of their equipment had not been designed with this in mind.

*The [adult thermometer] reads under the tongue. Which I think is horrifically invasive*,* especially when someone is in labor. Like just a really unpleasant thing. They take ages*,* they take well over a minute. And when you’re in labor*,* in particular*,* that’s really vile.*

Midwife D.

Some midwives interviewed shared an awareness that the equipment they used could contribute to patients’ negative feelings about increased BMI and were keen to avoid this.

*Having to change the [blood pressure] cuff sizes for women with a larger BMI… I’m very aware that women with a larger BMI have clocked that we do it and can*,* you can see the instant awkward look. So that’s a shame… From that perspective it would be nice if there was something*,* you know*,* a generic one size fits all*,* if possible.*

Midwife A.

One of the midwives, who cared for a high number of non-English speaking women, felt that their carbon monoxide (CO) monitor was confusing to her patients, as the instructions for using it were complicated to explain, even with an interpreter. This monitoring is used to assess a woman’s CO exposure, and is intended to support referrals to smoking cessation services and provide motivation to those already quitting smoking.

### Theme two: equipment challenges working within the NHS

#### 2a. Lack of availability of appropriate equipment

All the participants reported having little or no involvement in any of the purchasing decisions for equipment; the introduction of new health technologies; or their Trust operating procedures and policies regarding the use of equipment, such as referral pathways. There was a consensus that many of the issues with current equipment were caused by Trusts generally opting for lower priced options, with little consideration given to the staff using it.

*It’s really hard to influence your purchasing*,* isn’t it? Because it’s all done on a mass level these days and they’re influenced by price.*

Midwife D.

Many of the midwives interviewed reported buying their own equipment, because they were not provided with something they felt they needed or because they felt that the items in their kit did not meet an acceptable standard. Examples included ophthalmoscopes, stethoscopes, thermometers, and oxygen saturation monitors.

*I bought my own stethoscope because the ones that we were provided with were pretty cheap and hurt my ears.*

Midwife C.

Several reported frustrations at having to share equipment with colleagues, such as baby weighing scales and blood pressure cuffs, despite the equipment being required frequently.

*Because you need to have a large cuff and a small cuff*,* but generally we’ve got one*,* and then if we need a large cuff we go and find that from somewhere else and take that out to a specific visit.*

Midwife B.

The midwives all used aneroid devices, not digital devices, for blood pressure monitoring. Some were interested in using digital monitors, but these were either not available or the midwives had been dissuaded by concerns regarding their accuracy. Some of the midwives felt that aneroid devices were quicker to use, whereas others felt that access to a digital device would free up time to complete another task.

*I have been in GP surgeries where there is the electronic [BP monitor] to hand. And then I find that if you use that*,* you almost inevitably go ‘That’s a bit high*,* let me actually check properly.’*

Midwife K.

Two devices that many of the midwives were expected to use often, but that were not readily available, were transcutaneous bilirubinometers (TCBs) (used to monitor newborn jaundice) and oxygen saturation monitors for babies (used during routine and emergency assessments for respiratory or cardiac complications). The midwives were sensitive to the fact that these items are expensive, but still found it challenging not to have easy access to them. They described considerable time spent traveling, collecting and returning the devices between appointments. Donations from charities were sometimes used to pay for equipment that NHS Trusts were unable or unwilling to supply.

*You could be travelling thirty miles to the hospital to get a TCB… it’s very frustrating. But the problem is*,* they’re four and a half thousand pounds each. We have just bought a couple more because someone donated money. In an ideal world*,* you’d have more. Each midwife would have one… Because there’s a lot of time wasted going looking around for one of these monitors. We do oxygen saturations on a baby as well. We have to go to the hospital to get that [monitor]*,* because there’s very few of those… which is again time-consuming as a community midwife*,* to go backwards and forwards all the time.*

Midwife H.

#### 2b. Lacking autonomy in how to utilise equipment

On the whole, the midwives felt that all of the tests they were expected to perform were appropriate and beneficial to the care they provided. One exception to this was CO monitoring. At the time of the interviews, and still now, Trusts were instructed to share compliance data as part of the Maternity Incentive Scheme [13]. While the majority of those interviewed were in favour of the availability of this test to support smoking cessation, several wanted autonomy to use their equipment as they saw fit, as opposed to following a prescriptive, target-driven approach.

*So people weren’t being screened [for CO]. But then well*,* the email [from senior leadership] said something along the lines of*,* “You’re not doing it at the 36 week appointments*,* so we’re going to make you do it at every appointment.” I don’t think that’s evidence-based. I think that’s sort of “you naughty midwives”*.

Midwife F.

### Theme three: areas of unmet needs

When questioned about areas of unmet needs, many of the midwives were hesitant about the idea of adding new tests, as they felt that this might overcomplicate care or deskill them, or the students they supported in practice. They were also concerned about the cost implications for their Trusts.

*It’s a fine balance between sort of introducing technology*,* because then does that make us more just obstetric nurses? Rather than the skills that you have*,* the skills of palpation. Obviously you do question yourself at times… “it breech? Is it something else?” But*,* that’s just wisdom you wouldn’t want to lose and technology doesn’t take into account everything.*

Midwife E.

*Midwives I know like to know their equipment and they’re a bit funny when it comes to change at the best of times. Also*,* the financial implications for the Trust securing the equipment*,* in my eyes would be a nightmare.*

Midwife A.

Several midwives expressed unease regarding introduction of equipment into the community that they were used to seeing in the hospital. While some felt that this approach would be beneficial, there was uncertainty about how it would work logistically, for example, how they would be trained and which pregnancy complications they could diagnose and manage without input from an obstetrician.

*I suppose I wouldn’t be keen to have the CTG in every birth centre because you need all the ongoing sort of support for that as well. It’s another skill that having worked in the community for such a long time you know*,* interpreting the CTG’s is difficult and it’s not something I have to do now.*

Midwife C.

Generally, when participants reported wanting more health technology, they described devices that they knew were already on the market and that were often already being used by community midwives in other Trusts (including those interviewed for this study). These included transcutaneous bilirubinometers, serum bilirubin measurement equipment, oxygen saturation monitors (adult and newborn), tests for amniotic fluid, and CTGs. There were two POCTs not currently being used by any of those interviewed which the midwives felt would be useful and practical to introduce to a community setting. These were portable ultrasound scanners for presentation scans and IVD POCTs for diagnosing anemia, preeclampsia, and infections such as Group B Streptococcus (GBS) and UTIs.

There were four main reasons the midwives gave with regard to why they might like to see new technologies introduced:

1) To support decision making. Some midwives felt that their assessments lacked the same components and, therefore, the completeness of a hospital assessment. Where relevant portable technology existed, they felt should be incorporated.

*I’ve worked with MSWs [maternity support workers] and I can see protein [showing as present on the urinalysis dipstick]*,* they can’t or vice versa*,* so actually*,* it it’s very subjective… I think we should use a urinalysis machine to analyse it rather than our eyes. That could be a really useful tool.*

Midwife K.

*A little pulse oximeter would be really handy to have in the kit bag… We do have to calculate the early warning score in the home*,* and obviously oxygen saturation [monitors] would help with that.*

Midwife L.

2) To reduce the number of hospital visits for patients. This was important to the midwives as they felt that women often struggled with getting to the hospital or would prefer to avoid the hospital for psychological reasons.

*In the main hospital*,* they will do PROM [pre-labor rupture of membranes] testing but for some reason at the midwife-led unit we don’t have it… We then have to send them [the women] all the way up to the hospital. The same with women who’ve a planned home birth. They don’t want to have to come into hospital.*

Midwife J.

3) To improve workload. For example, not having to spend time (often outside of normal working hours) transporting samples to a hospital or waiting for results to arrange appropriate treatment. Additionally, the workload of hospital colleagues could be reduced.

*I’d like better access to testing. Just the whole thing about having to keep going back to the hospital with everything*,* that’s my big bugbear… I would like to be able to get an instant result on a swab for Group B Strep. That would be really useful. The fact that they take such a long time to come back. An MSU [mid stream urine culture] can take 5 days before I get the result*,* or a swab*,* that is ridiculous to me.*

Midwife M.

*One idea for the future*,* that I know we have mooted round in the office*,* is a handheld one [ultrasound scanner] that you can almost sort of see the images on a mobile phone*,* just to do a basic presentation scan. Because it is difficult sometimes for capacity for scanning*,* because they’re so busy with obviously dating scans*,* anomaly scans*,* routine growth scans.*

Midwife E.

4) To reduce the overmedicalisation of care. The midwives felt that once in the hospital setting, women referred for one concern might inadvertently be screened for other potential risks, which might heighten anxieties around birth and lead to a cascade of interventions.

*I’d like one of those little handheld ultrasound machines*,* just so I can check if babies are breech. I don’t want to get involved in size… But I’d just like*,* you know those ones that you*,* you’re like ‘I can’t be 100% sure’ and you send them for a scan at 36 weeks and then something else gets picked up that’s a load of old rubbish.*

Midwife D.

## Discussion

This is the first qualitative study in which UK based community midwives were asked about the health technology they use to support their role. This study set out to understand more about what midwives think of their existing health technologies as well as what they would like to see in the future. This included considering any diagnoses that they cannot currently make in the community but that they felt would be of benefit to their patients or themselves. The research captured not only practical issues with equipment but also the midwives’ emotional and professional responses within the NHS context.

### Summary of key findings

Midwives are generally satisfied that they are meeting the needs of the women in their care with the POCTs and equipment currently available to them. The findings reveal the challenges faced by community midwives working within the NHS, including a lack of involvement in purchasing decisions, frustrations about having to share or buy their own equipment, and the desire for autonomy in using equipment. The perceived tendency for Trusts to opt for lower-priced options was criticized for failing to consider the needs of staff. A recently published qualitative study investigating UK community midwives’ experiences providing postnatal care corroborates the finding that midwives were dissatisfied with the limited availability of resources [14]. The midwives described spending a considerable amount of time in their working day getting access to shared equipment [14]. This is concerning, as work frustrations have been linked to the high levels of work-related burnout, depression and anxiety observed in UK midwives [15].

The midwives had a woman centred approach to their practice and were thoughtful about ways in which equipment may negatively impact how women felt. They were keen to have a flexible, individualized approach to equipment usage as opposed to strict, target-driven protocols. This tension was particularly evident in discussions around CO monitoring. The midwives discussed the need for blood pressure cuffs of different sizes and were concerned about how this might make women feel. Marginalized groups, including those with a raised body mass index (BMI), can find accessing healthcare particularly stressful and might avoid seeking help if they feel any shame about their size [16].

Midwives have some interest in the introduction of new equipment, particularly where it may support their decision-making, improve their workload, and reduce the number of women needing to go to the hospital, which they feel can be logistically challenging and can lead to a cascade of unnecessary medicalisation. At the same time, they do not want to see community-based midwifery units being turned into ‘mini’ obstetric units. A qualitative study exploring the experiences of midwives and students in a midwifery led unit found similar themes to this work and community midwives’ value of their tacit knowledge and the ability to manage pregnancy and birth in the absence of technology [17]. When introducing new POCTs in community maternity settings, it is important to understand that the differing experiences and beliefs of staff will impact how able and willing they are to assimilate technology into their role.

The midwives noted several specific POCT devices, such as portable ultrasound scanners and In vitro diagnostic (IVD) POCTs, that they believe would be beneficial in their practice. The perceived advantages of these devices were mainly related to reducing the need for hospital referrals and providing immediate reassurances or treatments to their patients. At the same time, the midwives showed apprehension toward incorporating ‘hospital equipment’ into their community settings. They were concerned about the practicalities of this, such as referral pathways or training potentially not meeting their needs. This indicates the necessity of careful planning when introducing new equipment, ensuring that support is readily available to promote the correct usage and interpretation of the results from these devices.

### Strengths and weaknesses of this study

The strengths of this study include the use of semi-structured interviews, which enable an in-depth exploration of the varying experiences of a range of participants. The midwives interviewed included those working in rural, suburban, and inner-city locations in England and Wales. There was a large range in the number of years working as a midwife, from newly qualified to more than 20 years. Most of the midwives had worked in the hospital, as well as in the community, with some having a shared role between the hospital and community.

The broad exploratory base of the study meant that we did not discuss individual tests in great depth. Understanding of specific health technologies, as well as specific clinical presentations and contexts, could be the focus of further research. All participants were from England and Wales. As such, our findings are applicable to English and Welsh stakeholders and clinicians and may not be transferable to other settings.

### Future research

Increasing the use of health technology is part of NHS strategies to increase the number of medical conditions managed outside of the hospital setting. Research will be needed to test the acceptability and effectiveness of current and new devices, within the maternity setting. Those looking to implement novel POCTs will need to consider the unique context of pregnancy and birth (such as over-medicalisation) and the physiological changes that occur in pregnancy (such as a shift in ‘normal’ ranges for some blood test results). This will include understanding more about pregnant women’s and new parents’ views and experiences of POCTs. The challenges of using equipment with non-English speaking patients were highlighted, making it important to ensure that the experiences of these women are included, as well as those from other minority groups.

Although not discussed in detail, this study revealed a lack of equity in the equipment used by the midwives interviewed from different services. Medical technologies that some of the midwives flagged as an unmet need were often already being used by community midwives in other NHS Trusts interviewed in this study. This disparity did not appear to be related to differences in work settings, such as proximity to a maternity hospital. For example, in some NHS Trusts, midwives are trained and have the equipment needed to test babies for jaundice using transcutaneous monitors and blood testing [serum bilirubin (SBR)]; others are only able to perform a transcutaneous reading, with babies who require subsequent blood testing being referred to the hospital; and some simply refer all the babies who appear jaundiced to a hospital to be tested there. More focused enquiry is needed to understand the reasons for these differences and the effects that they have on patient experience and health outcomes, particularly for women from lower socio-economic groups, where travel may be challenging, and for babies from non-white backgrounds, where visual inspection for jaundice is known to be less accurate [18].

## Conclusion

This study demonstrated that there are several areas of unmet needs for community midwives that should be investigated, but arguably more pressing is improving the availability and quality of existing technologies. NHS England recommends that digital blood pressure devices should replace aneroid devices as the diagnostic screening tool in maternity services [19]. This guidance was released after the interviews for this study were completed. At the time of the interviews, aneroid devices were widely used and there were concerns raised by the community midwives about the accuracy of digital devices. In the author’s (HE) NHS Trust, digital devices were made available to community midwives in early 2024.

The following criteria should be considered when designing or procuring diagnostic technology for use in maternity care, based on the results of this study:

1. Will this product improve clinical decision making; reduce hospital-based care; or support clinician time management?
2. Has this product been designed specifically for a maternity population? Could the use of this product cause any physical or psychological harm, or disturb physiological labor?
3. Is the quality sufficient to meet the demands of transportation, changes in temperature and frequent use in a variety of environments?
4. Is this product at a price point that ensures that the midwifery workforce can be adequately equipped?
5. Does this product come with sufficient evidence of accuracy and reliability in the maternity population to ensure that the midwives trust the results given? Is there evidence of when it is cost effective to use this diagnostic?
6. Are there evidence-based guidelines to support midwives in their clinical decision making when using this product?

## Data Availability

The datasets during and/or analysed during the current study available from the corresponding author on reasonable request.

## Abbreviations

BMI: Body Mass Index
CO: Carbon Monoxide
CTG: Cardiotocography
DIY: Do it Yourself
IVD: in vitro diagnostic
NHS: National Health Service
POCTS: Point of Care Tests
SBR: Serum Bilirubin
SROM: Spontaneous Rupture of Membranes
TCB: Transcutaneous Bilirubinometer
UTI: Urinary Tract Infection
